# Mental health priorities and cultural-responsiveness of the Mental Health First Aid (MHFA) training for Asian immigrant populations in Greater Boston, Massachusetts

**DOI:** 10.1186/s12888-024-05894-x

**Published:** 2024-07-16

**Authors:** Min Kyung Kim, Grace S. Su, Angel N.Y. Chan, Yuxin Fu, Yanqing Huang, Chien-Chi Huang, Ben Hires, MyDzung T. Chu

**Affiliations:** 1 Tufts Clinical and Translational Science Institute, Boston, MA USA; 2 Boston Chinatown Neighborhood Center (BCNC), Boston, MA USA; 3 Asian Women for Health (AWFH), Boston, MA USA; 4 Institute for Clinical Research and Health Policy Studies, Tufts Medical Center, 35 Kneeland Street, Rm. 1004, Boston, MA 02111 USA

**Keywords:** Asian mental health, Mental health training, Mental Health First Aid, Community based participatory research, Cultural responsiveness, Cultural appropriateness, Greater Boston

## Abstract

**Background:**

Asians and Asian Americans have the lowest rate of mental health service utilization (25%) in the US compared to other racial/ethnic groups (39 − 52%), despite high rates of depression, anxiety, and suicidal ideation. The lack of culturally-responsive mental health trainings hinders access to mental health services for these populations. We assessed the mental health priorities of Asian communities in Greater Boston and evaluated cultural responsiveness of the Mental Health First Aid (MHFA), a first-responder training teaching participants skills to recognize signs of mental health and substance use challenges, and how to appropriately respond.

**Methods:**

This is community-based participatory research with the Boston Chinatown Neighborhood Center (BCNC), Asian Women For Health (AWFH), and the Addressing Disparities in Asian Populations through Translational Research (ADAPT) Coalition. We conducted focus groups with community-based organization staff and community members to assess mental health priorities of Asian populations in Boston, MA. We then evaluated the utility and cultural-responsiveness of the English-language MHFA for Asian populations through pre- and post-training questionnaires and focus groups with community participants. Paired t-tests were used to evaluate questionnaire responses. Thematic analysis was used to analyze interviews.

**Results:**

In total, ten staff and eight community members participated in focus groups, and 24 community members completed the MHFA and pre- and post-training questionnaires. Common mental health challenges in the Asian communities reported by participants were loneliness, high stigma around mental illnesses, academic pressure, and acculturation stress. Compared to pre-training, MHFA participants demonstrated lower personal mental health stigma (*p* < 0.001) and higher mental health literacy (*p* = 0.04) post-training. Participants also noted the lack of data statistics and case studies relevant to Asian populations in the training, and desired the training be offered in languages spoken by Asian ethnic subgroups (e.g., Chinese, Vietnamese).

**Conclusion:**

Cultural-responsiveness of the MHFA for Asian populations could be improved with the inclusion of data and case studies that capture common mental health challenges in the Asian communities and with translation of the MHFA to non-English languages predominant in Asian communities. Increasing the cultural relevance and language accessibility of the MHFA could facilitate wider adoption of these trainings across communities and help to reduce mental health stigma and gaps in literacy and service utilization.

**Supplementary Information:**

The online version contains supplementary material available at 10.1186/s12888-024-05894-x.

## Background

Nationally, Asians and Asian Americans (hereafter referred to as Asians) have the lowest rate of mental health service utilization (25%) in the US compared to other racial/ethnic groups (39 − 52%), despite high rates of depression, anxiety, and suicidal ideation [1–3]. Moreover, with the COVID19 pandemic, Asian communities have experienced significant increases in mental health issues [4, 5]. From 2019 to 2020, depression diagnoses increased by 104% and anxiety disorders by 97% for Asian populations in the US [6]. In 2021, more than 4 in 10 Asian persons reported current mental health symptoms [7], and one in every six Asian adults reported experiencing a hate crime or hate incidence [8–10].

In Greater Boston, mental health continues to be a high priority in Asian communities [11]. In 2021, approximately 11.6% of Asian students in Boston seriously contemplated suicide [12]. In addition, recent studies highlight increasing financial insecurity, workplace health and safety concerns, crowding, racial discrimination [13], and problem gambling [14] in Asian communities throughout Greater Boston. These mental health risk factors are associated with increased symptoms of anxiety and depression, diminished life satisfaction, and suicidal ideation [4, 14]. Despite these challenges, Asians have the lowest rates of mental health service utilization [15, 16], with significant barriers to access such as the lack of culturally-responsive and linguistically-accessible providers, long wait lists for care, and stigmatization [3, 11]. In addition, the model minority myth, which portrays Asians as a monolithic and universally healthy group, reinforces poor data collection and representation of mental health challenges in the Asian communities, which then leads to limited funding and mental health services dedicated to these communities [5, 17].

One approach to increase the availability of culturally-responsive mental health services is through partnerships with community-based organizations (CBOs). CBOs serve as cultural liaisons in underserved communities and provide essential, linguistically and culturally-appropriate care to clients whose needs are often unmet in mainstream services [14, 18]. Throughout the pandemic, CBOs have had to rapidly respond to increasing demands for mental health support services among their clients [13, 19, 20]. For instance, the Orange County Asian Pacific Islander Community Alliance’s staff and their community health workers provided mental health workshops to support Asian students and families through virtual education sessions and mental health telehealth services during the COVID-19 pandemic [21, 22]. However, lack of funding and workforce with culturally competent mental health training has hindered CBO’s capacity to sufficiently respond to the growing demand for mental health services. In addition, most existing community-level trainings are not culturally-tailored or linguistically accessible to Asian populations.

A widely recognized community-level mental health training is the Mental Health First Aid (MHFA). The MHFA is a mental health first-responder program that teaches community members about risk factors and warning signs for poor mental health and substance use concerns, and strategies to appropriately help someone in both crisis and non-crisis situations [23]. The MHFA training has been implemented in several Asian immigrant communities globally (e.g., Chinese and Vietnamese) and has shown to improve mental health literacy and stigma [24–26]. Among a Chinese community in Australia, the MHFA training improved the level of mental health literacy and reduced stigmatizing attitudes [24]. However, it has not been implemented in Asian immigrant communities in the U.S. Also, at the time of our study, it was only available in English and Spanish languages.

To better understand whether the MHFA training can be utilized as community-based training for Asian populations in Greater Boston, MA, we evaluated the utility and cultural-responsiveness of the MHFA training to address mental health conditions and care needs for Asian populations in Greater Boston. We also conducted focus groups with staff and peer educators from two local CBOs to better understand the context of mental health among Asians in Greater Boston.

## Methods

### Study design

This study employed a community-based participatory research (CBPR) approach [27] in collaboration with the ADAPT (Addressing Disparities in Asian Populations through Translational Research) Coalition at Tufts Clinical Translational Science Institute (CTSI) and two CBOs: the Boston Chinatown Neighborhood Center (BCNC) and Asian Women for Health (AWFH). ADAPT is a community-academic coalition based in Boston’s Chinatown [28], of which BCNC and AWFH have been core community partners of since 2011. BCNC provides a range of family-centered programs to approximately 13,000 children, youth, and adults from Greater Boston including Chinatown, Quincy, and Malden, MA each year [19]. AWFH is a peer-led, community-based network dedicated to advancing Asian women’s health and wellbeing across 11 partner sites in Boston’s Chinatown, Somerville, Dorchester, Roxbury, and Worcester, MA [20]. The objectives of this study are aligned with BCNC and AWFH’s programmatic goals to address community mental health needs in Asian communities. This study received the Institutional Review Board (IRB) approval from Tufts Medical Center/Tufts University (reference number: 00003155).

### MHFA training

The MHFA training program is an evidence-based curriculum that teaches participants risk factors, warning signs, and symptoms of various mental illness (e.g., depression, anxiety, trauma, psychosis, substance use disorders, self-injury, suicidal behaviors). The program has more than 750 instructors delivering trainings globally, and organizations in 14 countries adopting the program [24]. More information about the MHFA has been previously described [29]. The synchronous instructor-led component includes interactive learning activities such as case-based learning, role play, and self-reflection. In addition, participants learn a structured response, e.g., ALGEE, consisting of five actions: (1) **A**ssess risk of suicide or harm; (2) **L**isten non-judgmentally; (3) **G**ive reassurance and information; (4) **E**ncourage the person to get appropriate professional help; and (5) **E**ncourage self-help strategies.

### Delivery of the training

In 2022, two staff from each organization received the Youth and Adult MHFA instructor trainings, respectively. The *Youth MHFA* training teaches participants how to help adolescents (age 12–17 years) experiencing mental health challenges [30], and the *Adult MHFA* training teaches participants how to help adults (age 18+ years) [31]. After being qualified as MHFA instructors, BCNC facilitated three Youth MHFA trainings via blended (i.e., 2 h of self-paced online learning and 6 h in-person training) and online formats from December 2022 to May 2023 with 41 participants. AWFH facilitated two adult MHFA trainings via an online format in January 2023 with 8 participants. In total, 49 community members participated in the MHFA trainings.

### Study populations

The study population included two target groups:

1. *CBO staff and peer educators for focus group discussions.* Eligibility criteria for participation included: affiliation with either BCNC or AWFH, at least 18 years old, and work/interaction with Asian populations in the areas of mental health. Two virtual focus groups were facilitated - one for each organization. Each focus group included five staff and lasted approximately 60 min. After the discussion, each participant received $20 electronic gift cards for their participation.
2. *Community participants who completed the MHFA training for structured questionnaire and focus group discussions.* Eligibility criteria included: affiliation with Asian communities in the Greater Boston area, at least 18 years old, and enrolled in either BCNC or AWFH’s MHFA training. Recruitment for community participants occurred after they registered for the MHFA training. Participation in the research study was voluntary and separate from MHFA training. Participants were asked to complete (1) an online survey (about 10-minutes long) before (pre-) and after (post-) the MHFA training, and (2) a virtual focus group discussion (about 60 min). If participants completed the pre- and post-MHFA questionnaires, they received a $15 electronic gift card. Those who participated in the focus groups received an additional $15 electronic gift card for their participation. In total, there were 8 community participants in the focus group.

### Data collection

#### Focus group discussion

We facilitated focus group discussions with CBO staff and peer educators to learn more about mental health conditions and care needs for Asian populations in the Greater Boston area. We asked five open-ended questions about Asian mental health needs; common mental health seeking behaviors; training and education needed to support Asian community members with mental illnesses; and cultural, community, familial, language, and immigrant-related contexts to consider when working with Asian community members.

We also facilitated focus group discussions with community participants who completed the MHFA to learn more about the utility and cultural responsiveness of the MHFA training. We asked five open-ended questions about common mental health conditions and challenges among Asian peers; the most and least relevant parts of the MHFA training for Asian populations; specific Asian communities that can benefit from the training; and any recommendations for MHFA trainings to be culturally responsive for Asian populations.

#### Pre- and post-training questionnaires

The pre-MHFA online questionnaire was sent to participants one week prior to their MHFA training date. The questionnaire consisted of 69 questions across seven sections (see Supplemental file 1), comprising of the following:

1. Demographics (e.g., race/ethnicity, nativity, age).
2. Self-reported health. Participants were asked “In general, would you say your health is?” with five response options (excellent, very good, good, fair, and poor).
3. Potential barriers to mental health care based on the Barriers to Access to Care Evaluation (BACE) Scale [32]. Participants were asked to think of an Asian person they knew/were close to who has/had a mental health problem when responding to the following question, “Have any of these issues ever stopped, delayed, or were discouraged this person from getting or continuing with professional care for a mental health problem?” based on 15 identified barriers (e.g., lack of health insurance) and using a four-point Likert response scale (0 = not at all to 4 = a lot).
4. Perception of mental health help-seeking behaviors for Asian persons with mental health issues. Participants were asked how likely an Asian person they knew/were close to with a mental health problem would seek help from a list of 12 resources/services (e.g., intimate partner, traditional healer) using a three-point Likert scale response (0 = not likely to 2 = very likely).
5. Availability of mental health supports or services in the local community and whether they were linguistically-accessible and culturally-responsive for the Asian populations, such as: “Is this service provide in language accessible to Asian non-English speakers?“; “Does this service have culturally-competent providers who have worked with Asian patients before?“) with binary response options (yes/no) based on help-seeking questionnaire [33, 34].
6. Personal and community-level stigma to address mental health issues based on a vignette of a subject named Kim with depression [35, 36]. The same vignette was used for participants in the adult and youth MHFA trainings, but the subject’s age varied (e.g., a 30-year old adult and versus a 15-year old teenager, respectively) (see section V in Supplemental file 1 and section I in Supplemental file 2). To measure personal stigma, respondents were asked, “*Please indicate how strongly YOU PERSONALLY agree or disagree with each statement*,” by rating 9 statements (e.g., people with problems like Kim could snap out of it if they wanted) using a five-point Likert scale (1 = strongly disagree to 5 = strongly agree). Personal stigma questions were asked pre- and post-training to assess for potential changes. To measure Asian community stigma, respondents were asked to respond from the perspective of an Asian/Asian American community (“*Please indicate how MOST PEOPLE in the ASIAN/ASIAN AMERICAN COMMUNITY agree or disagree with each statement*”, to each of the 9 statements (e.g., most people in the Asian community believe that people with problems like Kim could snap out of it if they wanted) using a five-point Likert scale. Community-level stigma was only asked pre-training to understand the overall stigma among Asian community members. Scores were summed and averaged across 9 statements, with low scores reflecting less stigmatizing attitudes.
7. General mental health literacy [35] and mental health literacy specific to Asian populations [35, 37–40]. The participants were asked to rate whether six knowledge-based statements on mental health issues in the general population (e.g., *Around half of mental disorders starts during childhood or* adolescent) and six statements on mental health issues specific to Asian populations (e.g., *Suicide is the leading cause of death for Asian youth ages 15–24 years old*) were True or False. Responses were scored as follows: correct (+ 1), incorrect (-1) and don’t know (0). Scores were summed such that higher scores reflect higher literacy about mental health. The maximum possible score was 12.

Immediately following the completion of MHFA training, participants were sent the post-MHFA online questionnaire, which consisted of 32 questions (see Supplemental file 2). The three sections on demographics, self-reported health, and Asian community stigma from the pre-training questionnaire were excluded. Also, for the section on potential barriers to mental health care, the question was modified in the post-training questionnaire to ask whether common potential barriers were covered in the MHFA training (e.g., “Did the MHFA training that you just completed help you recognized any of the following potential barriers for individuals seeking mental health care? Select all that apply from a list of 19 barriers (e.g., lack of insurance coverage, concern that people may find out, fear of medication side effects). The post-training questionnaire also asked participants for free-text feedback about cultural competence of MHFA training to Asian and immigrant populations (e.g., “During the MHFA training, were there any specific examples or case studies that were tailored to Asian populations?”).

### Statistical analyses

#### Qualitative analysis

Thematic analysis in five phases was used to analyze the focus group data [41]. The first phase involved two reviewers familiarizing themselves with the data through repeated reading and transcription. The second phase involved two reviewers independently generating initial codes of the entire data. During the third phase, two reviewers searched for themes via reviewing, defining, and naming themes. During the fourth phase, all themes were reviewed by discussing the relationship among the themes, presenting them in a more systematic way as a map/figure, and refined themes (e.g., merging subthemes that have overlapping meanings). In the case of differences in coding, the two reviewers met to discuss the themes and come to a consensus on one theme and/or include as a subtheme. Phase five involved writing and preparing a manuscript, including selecting quotes that represent the essence of each theme.

#### Quantitative analyses

Descriptive statistics of the study population were assessed. Mean and standard deviation of personal and Asian community stigma and mental health literacy were calculated. Paired t-test, commonly used to compare the average score of two paired samples, was used to compare personal versus Asian community stigma from the pre-training questionnaire, and changes in personal stigma, mental health literacy from pre-training to post-training. Percent response rate was calculated for (1) literacy on the needs and barriers to mental health care self-reported by participants, (2) mental health help-seeking behavior for Asian members, and (3) availability of mental health services in Asian communities. All analyses were conducted in Stata SE v17.0 [42] and statistical significance was set at an alpha-level of 0.05.

## Results

### Demographics of study participants

In total, ten CBO staff and peer educators and eight community participants participated in the focus groups (Table 1). In addition, 24 community participants (BCNC: *n* = 21, AWFH: *n* = 3) completed the pre- and post- MHFA training questionnaires (Table 2). The majority (91%) of participants identified themselves as Asian, non-Hispanic, and primarily of Chinese ethnicity (Table 2). About half of participants were foreign-born (50%) and 72% of participants had at least a Bachelor’s degree.

**Table 1:** Study design and number of participants

	*N* (%)	Asked about mental health needs in local Asian communities?	Asked about utility and cultural responsiveness of MHFA?
**Focus groups**			
CBO^a^ staff and peer educators	10 (53%)	Yes	No
Community participants	8 (47%)	Yes	Yes
**Pre- and post-training questionnaires**			
Community participants	24 (100%)	Yes	Yes

Footnote: ^a^Community-based organizations: Asian Women For Health (AWFH), Boston Chinatown Neighborhood Center (BCNC). Ten staff and peer educators from AWFH and BCNC (five from each CBO) participated in the focus group discussions

**Table 2:** Demographics of community participants in the MHFA training (*N* = 24)

	*N* (%)
**Number of community participants**	
Adult MHFA– AWFH^a^ sponsored	3 (13%)
Youth MHFA – BCNC^b^ sponsored	21 (87%)
**Gender**^**c**^ (self-identified)	
Female	16 (76%)
Another gender (i.e., male, non-binary, agender)	5 (24%)
**Age**^**c**^ (range, in years)	22–66
**Race/Ethnicity** (self-identified)	
Chinese, non-Hispanic	16 (67%)
Another Asian, non-Hispanic (e.g., Filipino/Filipina, non-specify Asian)	6 (25%)
Another race/ethnicity (e.g., White, Black)	2 (8%)
**Nativity**	
US-born	12 (50%)
Foreign-born	11 (46%)
Missing	1 (4%)
**Self-reported health**	
Excellent	2 (8%)
Very good	13 (54%)
Good	7 (29%)
Fair	2 (8%)
**Education** ^**c**^	
High school and/or Associate degree	6 (29%)
Bachelor’s degree	6 (29%)
Graduate degree	9 (43%)

Footnote: <5 indicates the number of responses less than 5. Community-based organizations: ^a^Asian Women For Health (AWFH), ^b^Boston Chinatown Neighborhood Center (BCNC). ^c^Data for gender, age, and education level were only available for 21 participants who completed the BCNC training

### Context of mental health needs among Asian populations in Greater Boston

#### Focus groups

**Fig. 1:**
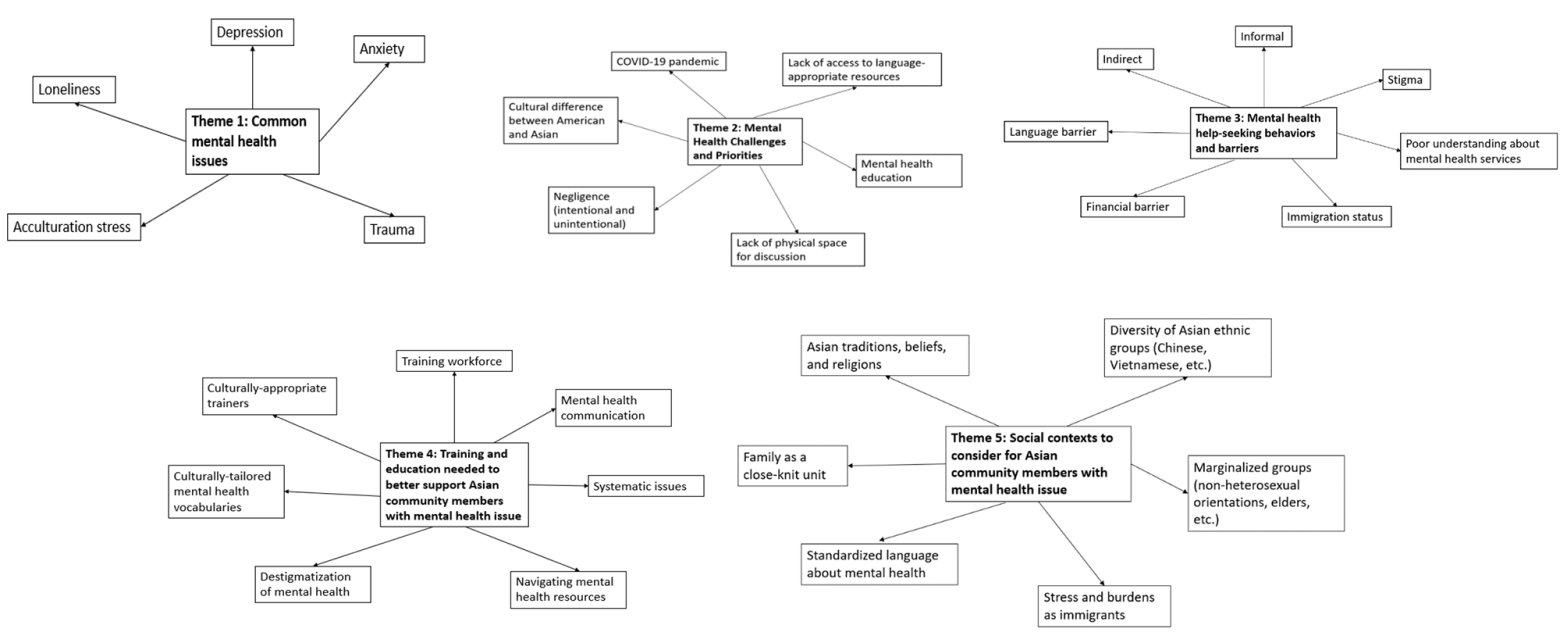
Thematic mapping from focus groups with *staff and peer educators* from two community-based organizations to assess mental health priorities in the local Asian communities (*N* = 10). Footnote: We facilitated focus group discussions with staff and peer educators from two community-based organizations to learn more about mental health conditions and care needs for Asian populations in the Greater Boston area. We asked five open-ended questions about Asian mental health needs; common mental health seeking behaviors; training and education needed to support Asian community members with mental illnesses; cultural, community, and/or familial contexts; and language or immigrant-related contexts to consider when working with Asian community members

**Fig. 2:**
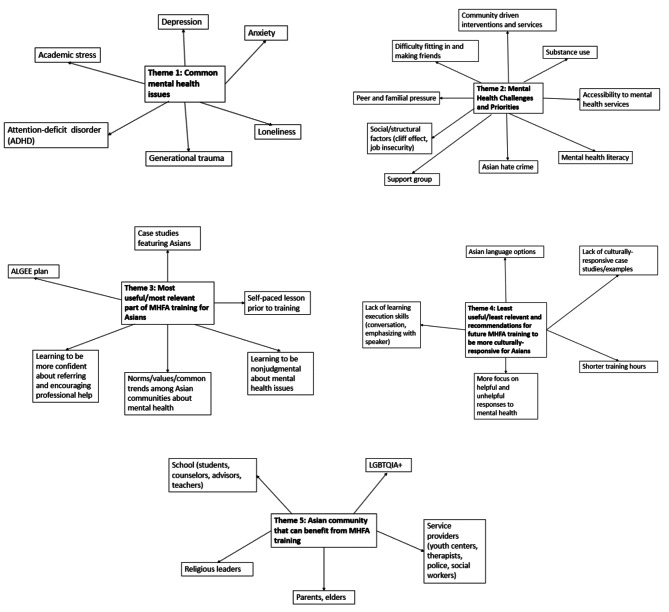
Thematic mapping from focus groups with *community participants* assessing mental health priorities in the local Asian communities, and the utility and cultural-responsiveness of the Mental Health First Aid training (*N* = 8). Footnote: We facilitated focus group discussions with community participants who completed the Mental Health First Aid (MHFA) training to learn more about its utility and cultural-responsiveness for Asian populations. We asked five open-ended questions about common mental health conditions and challenges among Asian peers; the most and least relevant parts of the MHFA training for Asian populations; specific Asian communities that can benefit from the training; and any recommendations for MHFA trainings to be culturally-responsive for Asian populations

Focus groups with CBO staff and peer educators are summarized in Fig. 1 and Supplemental Table 3. Focus groups with community participants are summarized in Fig. 2 and Supplemental Table 4. Overall, five major themes were discussed in the focus groups: (1) *Common mental health issues*, (2) *Mental health challenges and priorities*, (3) *Mental health help-seeking behaviors and barriers*, (4) *Training and education needs*, and (5) *Social-contextual factors*. They are described below:

*Theme 1: Common mental health issues*. CBO staff and peer educators identified the COVID-19 pandemic as a major factor in the increase in mental health conditions and challenges among Asian community members in recent years:

*“We’re hearing a lot about isolation, feeling lonely, among mostly elders as well as older adults who are kind of homebound during COVID*.”

*“The anti-Asian hate crimes for the past couple of years, a lot of them [e.g. Asian residents] are just terrified*.”

Other common mental health issues were loneliness, depression, anxiety (e.g., social, financial difficulty), trauma (e.g., immigration, generational), and acculturation stress:

*“People whose parents were immigrants have that survival mentality, and I think that feeds into their children having a lot of anxiety, depression, and being unable to navigate the world*”.

In addition, community participants shared that academic stress, depression, anxiety, loneliness, attention-deficit/hyperactivity disorder (ADHD), and generational trauma were identified as common mental health issues among their Asian peers:

“*Very often I feel that parents are projecting their life values, such as becoming a doctor or becoming a lawyer or getting married, and these projecting of life values can have a huge toll on the childre*n.”

*Theme 2. Mental health challenges and priorities*. CBO staff and peer educators identified mental health challenges and priorities such as the lack of access to language-appropriate resources; American versus Asian cultural differences in gender expectations and parent-child communication; lack of personal space and conflicting environments between home and school:

“*Even the topic of talking about mental health and talking about therapy is also difficult, too, because they [parents] don’t fully understand what that means or how to make their children feel loved and supported*.”

Other challenges raised were intentional negligence due to long wait times (e.g., deferring mental health care for other priorities), unintentional negligence (e.g., unawareness of mental distress), limited mental health education, and lack of physical recreational spaces to relieve mental distress:

“*You know [Asian Americans adults] are not going to go to a bar or have drinks with their friends. They want to go to karaoke or playing ping pong or something like that.”*

Community participants also identified the following mental health challenges for Asian populations: difficulty fitting in and making friends in America, substance use (e.g., marijuana), peer and familial pressures, and difficulty confiding personal issues to “strangers” (e.g., therapists). The increase in Asian hate crimes during COVID-19, limited accessibility to mental health services, poor mental health literacy, and loss of safety nets (e.g., cliff effect in public benefits, job insecurity, housing insecurity) were also important social and structural factors impeding access to mental health services:

*“Every time we make a referral, they will say, ‘Oh, the waiting is at least for six months or even more than a year,’ so it is very challenging for them to get services right away”*.

*“We’re kind of stuck on being poor all the time in order to be on MassHealth so we can pay, we can get medical coverage, our medication, see our therapists, our doctors”.*

*Theme 3: Mental health help-seeking behaviors and barriers*. CBO staff and peer educators shared that many of their clients used indirect approaches (e.g., engagement in family services or afterschool programs) and informal methods (e.g., online search, friends, religious leaders) to seek care options:

*“It’s very rare, at least based on my anecdotal experience, that clients come specifically to seek mental health assistance. It’s usually something that comes up in the discussion about other things [like family service, afterschool program]”*.

In terms of barriers to mental health help-seeking, staff and peer educators frequently mentioned stigma (e.g., worry about other members finding out) and the model minority myth:

*“One thing that’s unique in the Asian community is that we put less emphasis on individual than the fact we treat family as a unit, so there’s really not much privacy per say”*.

*“…mental health needs and distress are easily brushed off as a flaw of character”*.

Other common barriers included poor understanding about mental health services (e.g., limited language about mental health resources, mental health vocabularies, lack of trust in mental health system), financial barriers (e.g., high cost, prioritizing basic needs first), and language barriers (e.g., lack of Asian language speaking counselors and bilingual translators), and immigration status (e.g., documentation):

“*I’ve heard that a lot of undocumented folks are really afraid of seeking service, and when they are seeking services, it’s still hard for the provider to really build trust quickly, because [undocumented folks are] kind of afraid that things they get into or the services they seek will get them into trouble and I think that’s another thing to keep in mind.*”

*Theme 4: Training and education needs.* CBO staff and peer educators emphasized a great need for culturally-responsive mental health workforce training, communication strategies, and education to better support Asian community members with mental health issues:

*“A lot of the trainings I received I feel like were built for predominantly White culture”*

"*It’s important to have some type of training or education for the community about systemic issues in America, the injustices that’s happening to kind of break the myth, like the model minority myth.*”

Types of communication strategies suggested were more appropriate description of mental distresses and intergenerational dialogue between parents and children. Community participants also shared about the value of support and skills-based groups for residents to “come together and discuss self-help resources”, as well as community-driven mental health interventions and services.

*Theme 5: Social contextual factors.* Key social contextual factors to consider when addressing mental health issues within the Asian communities include: Asian traditional beliefs, religions, and medical practice; variations among Asian ethnic groups (e.g., Chinese vs. Vietnamese), and the incongruence with standardized mental health language (e.g., needing glossary for translation, lost in translation).

"*Different ethnic groups go to different treatment centers or community health centers*.”

*“I’ve had someone who actually told me about how her lungs are having too much heat. In [Asian] medicine, I think that’s relating to anxiety.”*

Other important contexts raised were the close-knit nature of the family unit and at the community-level that could contribute mistrust, lack of privacy, and high judgment; the stress and burdens that immigrants experience (e.g., traumatic, outsider/foreigner treatment); and within culture marginalization of specific groups:

"*The invisible judgment I get asked quite often like, is my neighbor going to see me talking to you?*”

*“[Immigrants] not having the life they used to have just because they moved to a different country, it’s sort of something that’s an ongoing struggle for them.”*

*“For a lot of gay and trans folks that are within the Asian community, they might face getting kicked out [of their homes].”*

### Questionnaire data

In the pre-training survey, community participants reported that the most common barriers to *seeking* care were stigma from family members (88%), concern that people might find out (79%), inability to describe or express one’s mental health issues (79%), and dislike of talking about one’s feelings, emotions, or thoughts (79%). In addition, participants reported structural barriers to *accessing* care, such as the lack of insurance coverage (74%) and financial costs (67%) (Table 3).

**Table 3:** Prevalence of barriers to mental health care and if these topics were covered in the MHFA training (*N* = 24)

	Identified by participants before the training^a^	Topics covered by MHFA training^b^
**Stigma**		
Concern about stigma from family member(s)	88%	88%
Feeling embarrassed or ashamed by community member(s)	71%	83%
Concern that people might find out	79%	83%
**Mental health literacy**		
Lack of knowledge on where to get the professional care	67%	83%
Lack of professionals from individual’s own ethnic or cultural group	63%	67%
Inability to describe or express one’s mental health issues	79%	67%
**Personal issues**		
Slow progress of mental health care	71%	50%
Preference to only seek help from family or friends	71%	46%
Unwillingness to improve	63%	21%
Preference to only seek alternative forms of help	54%	33%
Dislike of talking about one’s feelings, emotions or thoughts	79%	88%
**Social determinants of health**		
Lack of insurance coverage	74%	58%
Financial costs	67%	75%
Lack of transportation	54%	46%

^a^This is self-reported by participants on the pre-training survey. This question asked whether each listed potential barrier to mental health care ever stopped, delayed, or discouragedan Asian/Asian-American they knew with a mental health problem, with response options coded as: Not at all (0), Don’t know (0), A little [1], Quite a lot [1], and a lot [1]

^b^This is self-reported by participants on the post-training survey. This question asked participants to check off whether the Mental Health First Aid training help participants recognize any of the listed barriers. If the respondents checked the question, we coded as one [1]. If the box was not checked, we coded as zero (0)

Furthermore, participants’ perceived assessment of mental health stigma within the Asian community (mean 3.84, Standard Deviation [SD] 0.51) was more than two-folds higher than the level of stigma they would rate for themselves (mean 1.73, SD 0.70) (*p* < 0.001) **(**Table 4**)**.

**Table 4:** Mental health stigma and literacy scores pre- and post-MHFA training (*N* = 24)

	Pre-training scores	Post-training scores	*p*-value	
	Mean (SD)	Mean (SD)		
**Stigma** ^**a**^				
Personal	1.73 (0.70)	1.38 (0.44)	*< 0.001* ^*b*^	
Asian community	3.84 (0.51)	Not asked	NA	
	*< 0.0001* ^*c*^	NA		
**Mental Health Literacy** ^**d**^	5.96 (2.44)	7.17 (2.94)	*0.04* ^*e*^	

^a^Stigma for personal and Asian community was assessed using case vignettes via a scoring matrix: strongly agreed [5], agree [4], neither agree or disagree [3], disagree [2], and strongly disagree [1]. A higher score indicates higher stigma perception. Standard deviation (SD)

^b^Paired t-test of pre- and post-training surveys were used to calculate the *p*-value for the Personal Stigma category. *P* < 0.05 is considered statistically significant

^c^Paired t-test of pre-training responses for the Personal vs. Asian community categories was used to calculate this *p*-value. *P* < 0.05 is considered statistically significant

^d^Mental health literacy was based on 12 statements about mental health in the general population and in the Asian population, with a scoring matrix of: correct (+ 1), incorrect (-1), don’t know (0). A higher score indicates higher literacy about mental health. Maximum possible score was 12

^e^Paired t-test of pre- and post-training surveys were used to calculate the *p*-value for the Mental Health Literacy category. *P* < 0.05 is considered statistically significant

When asked about mental health help-seeking behaviors, the majority (88%) of participants identified both formal (e.g., mental health professionals) and informal (e.g., friend, colleagues, neighbors) types of outlets that Asian community members would seek out **(**Fig. 3a**)**. However, the *availability* of mental health services and supports in the local community was perceived to be low (mental health professional: 58%; community health center: 54%). In addition, the proportion of these services with in-language and culturally-competent providers for Asian, non-English speaking patients was also perceived to be low **(**Fig. 3b**)**.

**Fig. 3a:**
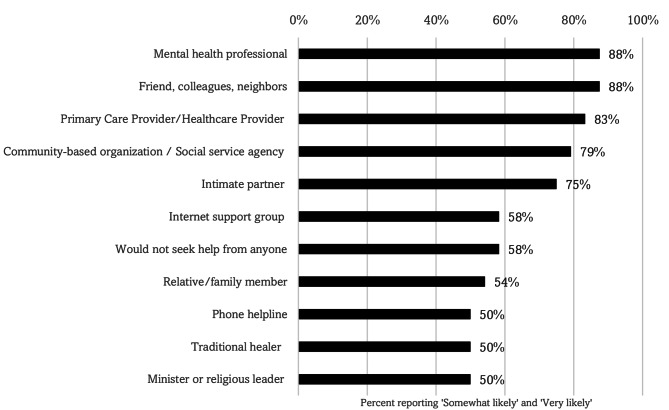
Prevalence of mental health supports or services likely sought out by an Asian/Asian-American person with a mental health problem (*N* = 24), Footnote: Questions asked about perception of mental health help-seeking behaviors for Asian persons with mental health issues. Participants were asked how likely an Asian/Asian-American person they know or are close to with a mental health problem would seek help from a list of 12 supports or services (e.g., intimate partner, traditional healer) using a three-point Likert scale response (0 = Not likely, 1 = Somewhat likely, 2 = Very likely). Responses of Somewhat and Very likely were then grouped together

**Fig. 3b:**
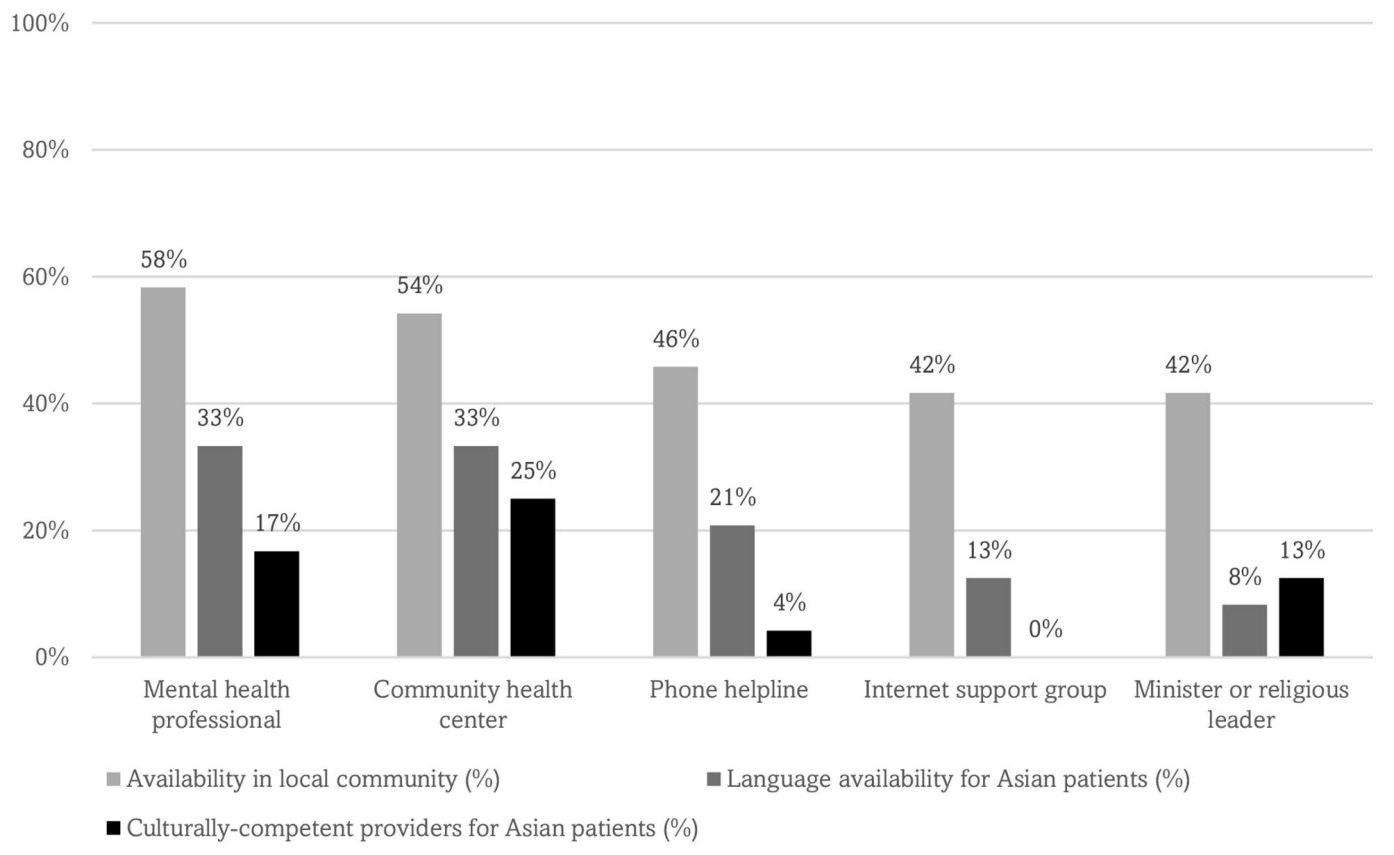
Prevalence of mental health supports or services available in the local community (*N* = 24). Footnote: Questions asked about the availability of mental health supports or services in the local community and whether they were linguistically-accessible and culturally-responsive for Asian populations, such as: “Is this service provided in language(s) accessible to Asian non-English speakers?“; “Does this service have culturally-competent providers who have worked with Asian patients?“) with binary response options (yes/no)

### Utility of the MHFA

Participants’ personal stigma about mental health issues significantly decreased from pre-training (mean 1.73, SD 0.70) to post-training (mean 1.38, SD 0.44) (*p* < 0.001). Also, participants’ mental health literacy improved from pre-training (mean 5.96, SD 2.44) to post-training (mean 7.17, SD 2.94) (*p* = 0.04) **(**Table 4**)**. In focus groups, community participants **(**Fig. 2, Supplemental Table 4) shared that the most useful and relevant parts of the MHFA training to address mental health issues for Asian populations were the ALGEE plan, self-paced lessons prior to training, and discussions about norms, values, and common trends among Asian communities related to mental health (e.g., high expectations/high functioning). After completing the MHFA training, community participants felt more non-judgmental towards individuals experiencing mental health issues (e.g., taking time to understand, not pushing them to make decisions). Participants also reported feeling more confident about referring and encouraging professional help among their Asian peers (Fig. 2, Supplemental Table 4).

Overall, almost all (92%) community participants said they would recommend the MHFA training to another community member (data not shown). In addition, they would recommend this training to other sectors and community stakeholders, such as school staff (e.g., counselors, teachers), service providers (e.g., youth centers, police, social workers), the LGBTQIA + community, parents and elders, and religious leaders (Fig. 2, Supplemental Table 4). However, participants shared general considerations for future MHFA trainings, such as having shorter training hours (e.g., workshop series, multiple days), more focus on helpful responses to mental health (Supplemental Table 4), expanding more on what it means to be culturally sensitive; and more coverage of topics such as providing psychoeducation about seeking professional help; dealing with a person who refuses help; parent-child communication role playing (Supplemental tables 5a & 5b).

### Cultural-responsiveness of the MHFA for Asian populations

At least two-thirds of participants reported that the MHFA training covered common mental health care barriers among Asian persons, such as stigma (e.g., concern about stigma from family members) and mental health illiteracy (e.g., lack of knowledge on where to get professional care). A few participants also found the case study featuring an Asian family helpful. However, barriers to mental health utilization related to personal issues, such as unwillingness to improve and preference to only seek help from family and friends, and the lack of insurance coverage were less likely to be covered in the MHFA, despite most participants identifying them as concerns in the Asian communities (Table 3).

Community participants also identified several limitations to the cultural-responsiveness of the MHFA for Asian and immigrant populations in the focus groups (Fig. 2, Supplemental Table 4) and post-training questionnaires (Supplemental tables 5a & 5b): the lack of learning execution skills (e.g., conversation, emphasizing with speaker); limited case studies/examples specific to the Asian and immigrant/refugee communities (e.g., immigrant status, family dynamic, academic stress); and the lack of language options. Specifically, 62% of MHFA participants reported that there were no examples or case studies tailored to Asian populations, while a third recalled only one or two instances. Similarly, the majority of participants (86%) reported no examples or case studies tailored to immigrant and refugee populations (Supplemental table 5a). In addition, community participants recommended that the MHFA training be provided in other non-English languages prevalent in the Asian communities, such as Chinese-Mandarin, Chinese-Cantonese, Korean, Japanese, and Vietnamese. Additional recommendations from participants are noted below:

*“I thought it would be useful to talk more about the stigma and how to have those conversations with different generations or people with very different views”*.

*“I think definitely the case study scenario discussion can include more cultural awareness, for example, based on new immigrants or undocumented immigrants.“*

*“…more on like dealing with academic stress and intergenerational struggle.“*

*“…getting more context as to why folks might be resistant to seeking help in regard to Asian cultures. I think more stress on not placing the blame on the individual links to that.“*

“*Discuss the concepts of shame, respect, and filial piety embedded in Asian culture and how they affect Asians at different stages in life.”*

*“Learning more about the history of those [immigrant and refugee] populations and why it may affect mental health.”*

## Discussion

Our study evaluated mental health priorities of Asian populations in the Greater Boston area and the utility and cultural-responsiveness of the MHFA training for Asian populations, particularly Chinese immigrants. We found that stigma against mental health care was perceived to be higher in the Asian community than at the individual level. The lack of culturally-responsive and linguistically-accessible mental health trainings, workforce, and resources were identified by community organizations and community participants as persistent barriers of mental health utilization in the Asian community. While the MHFA training improved mental health literacy and reduced personal stigma among Asian participants, the limited number of culturally-relevant case studies and language options beyond English and Spanish hindered its adaptability and linguistic accessibility to Asian populations, many of whom are immigrants and non-English speakers. Most studies of the MHFA training in Asian communities have been located outside of the U.S. and overall, have found demonstrated improvements in participants’ recognition of mental health disorders [24, 25, 43]. Our study is one of few studies to evaluate cultural relevance of the MHFA for Asian populations in the U.S. A prior study was conducted in Bhutanese refugee communities in Pennsylvania [25], however they found no changes in negative attitudes towards people with mental illness post-training in contrast with our study findings of improvements in personal stigma.

Our study is timely as mental health is a critical priority in the Asian communities and particularly in recent years since the COVID-19 pandemic [3, 7]. The Asian American Psychological Association reported that Asians who experienced anxiety or depression symptoms increased from 10% pre-pandemic to 40% during the pandemic [38]. However, systemic deficiencies in the number of culturally-responsive and linguistically-accessible mental health providers for Asian populations have created a growing chasm between demand and supply [3, 5]. As one potential alternative to narrow this gap, the MHFA training could be an accessible and timely way to train community members with skills and recognition to identify risk factors of poor mental health and appropriately intervene.

Cultural and immigration-specific contexts are important when addressing mental health care needs in Asian communities. Our findings in Greater Boston, particularly for Chinese residents, suggest that the MHFA curriculum may not adequately address the diverse mental health challenges among Asian, immigrant, and limited English-speaking populations in the U.S. Notably, a quarter of Asians in the U.S. (27%) live in multigenerational households [44]. Prior research suggests that Asian persons perceive mental health issues not at the individual level, but rather at the family level because the interdependent self is inseparable from other important relationships [45]. CBO staff, peer educators, and community members shared that the emphasis on filial piety in most collectivist Asian cultures often pose mental health challenges, such as academic stress and intergenerational struggles, that are distinct from those that would arise from the individualistic American culture.

In addition, language access was consistently named as a priority for CBOs and community participants in the Asian communities. Asian populations in the U.S. represent over 19 different ethnic groups and 10 different languages [46]. Asians also comprise the largest foreign-born populations in the U.S. [47], with only 57% being English-proficient [44]. Asians who do not speak English as their primary language often find comfort in seeking out others who speak their native language and can understand their cultural and familial perspectives [48]. As such, language inaccessibility poses a major barrier for MHFA participation for these populations. At the time of our study, only English and Spanish language versions of the MHFA trainings were available [23]. However, as of February 2024, the youth and adult MHFA curriculum is now provided in Chinese, Korean, and Khmer [49], which marks an important step towards increasing the MHFA’s accessibility and improving mental health equity for diverse Asian populations.

Furthermore, despite a reduction of personal stigma against mental health care observed among Asian participants in the MHFA training, community participants, staff, and peer educators from local CBOs still identified community stigma as the major reason for poor mental health service utilization in Asian communities. Our findings revealed that perceived mental health stigma in the Asian community was two-folds higher than personal stigma, reinforcing the need to address stigma at the community-level. In 2021, Asians showed the lowest utilization rate of mental health services (25%) compared to other racial/ethnic groups (36-52%) in the U.S [50]. Compounding this issue, the model minority myth perpetuates the misconception that Asians are inherently healthier than other racialized minority groups [17, 22]. As such, providing MHFA trainings to community members in collaboration with CBOs could be an effective way to address community-level stigma and foster supportive environments that encourage help-seeking behaviors and the receipt of mental health supports [22, 51].

### Recommendations and future directions

To address these unique challenges, it is important that the MHFA and similar mental health training resources be cultural-responsivene and linguistically-accessible to better address the diverse mental health needs and experiences in Asian communities. These resources should be made available in other languages predominant in Asian communities, such as Chinese (Cantonese and Mandarin), Hindi, Tagalog, and Vietnamese [44]. In addition, these resources should include health statistics and case examples that are specific to Asian populations as well as representative of the diverse ethnic groups and their cultural and immigration-related contexts. For example, Asian high school youth in the U.S. (29%) had the highest prevalence of suicidal thoughts and behaviors compared to the overall U.S. youth population (19%). Poverty rates in the U.S. vary greatly across Asian ethnic groups, ranging from 6% among Indians to 25% among Mongolian and Burmese groups [46]. In addition, discrimination against Asians and anti-Asian hate crimes have also substantially increased since the COVID-19 pandemic [52]. These population-specific case examples could be included in the MHFA curriculum to help trainees better contextualize and address these mental health inequities.

### Strengths and limitations

Our study included several strengths: First, we took a CBPR approach in collaboration with local CBOs, which promoted community empowerment, capacity-building, and co-learning [53]. Second, we incorporated a mixed-methods approach to assess mental health priorities among Asian populations and cultural-responsiveness of the MHFA to address these priorities. Third, our study contributes important insights about mental health needs, trainings, and supports for racially minoritized and underserved populations.

Our study also has several limitations. First, we recruited CBO staff, peer educators, and community participants from two local organizations that primarily served Chinese populations in Greater Boston. This approach may limit representativeness our findings to other Asian ethnic groups. In addition, the small sample size of our study may limit generalizability of findings. Second, our evaluation period was short-term, with the pre- and post-surveys administered within a week of the MHFA training. We did not assess whether findings were sustained over a longer period of time, and future studies could consider a longer follow-up period. Third, MHFA participants may have had a more positive perception towards mental health services and in effect, their response to the personal stigma question may be different from the general population.

## Conclusion

The MHFA training was found to be an effective tool at reducing personal stigmatizing attitudes towards people with mental illness and improving knowledge about mental disorders and appropriate response strategies to support persons in crises within the Asian communities in Greater Boston, MA. However, the cultural-responsiveness of the MHFA training for Asian populations can be improved by including more health statistics and case studies specific to these populations. Specifically, differences in cultural and immigration-related contexts across diverse Asian ethnicities need to be considered. In addition, more language options for the MHFA will increase its access and use among Asian and immigrant communities, which often have the lowest mental health service utilization despite the high prevalence of mental health challenges. Given that stigma against mental health care was percieved to be higher at the community-level than at the individual-level in Asian communities, collaborating with CBOs to offer community-wide mental health trainings could be an effective strategy to increase the utilization of mental health services.

### Electronic supplementary material

Below is the link to the electronic supplementary material.

Supplementary Material 1

Supplementary Material 2

Supplementary Material 3

Supplementary Material 4

Supplementary Material 5

## Data Availability

All the data supporting our findings have been presented in the manuscript; the datasets used and/or analyzed during the current study are available from the corresponding author on reasonable request.

## Abbreviations

CBO: community-based organization
MHFA: Mental Health First Aid
CBPR: community-based participatory research
BCNC: Boston Chinatown Neighborhood Center
AWFH: Asian Women for Health
IRB: Institutional Review Board
ALGEE: Assess risk of suicide or harm, Listen non-judgmentally, Give reassurance and information, Encourage the person to get appropriate professional help, Encourage self-help strategies
ADAPT: Addressing Disparities in Asian Populations through Translational Research, CTSI: Clinical Translational Science Institute
