# Supplementary material for: Mental health priorities and cultural-responsiveness of the Mental Health First Aid (MHFA) training for Asian immigrant populations in Greater Boston, Massachusetts
Source: BMC Psychiatry. 2024 Jul 16;24:506. doi: 10.1186/s12888-024-05894-x (PMC11251104; doi:10.1186/s12888-024-05894-x)
Supplement: Supplementary file 1 — Supplementary Material 1 [file 12888_2024_5894_MOESM1_ESM.docx]

Supplemental file 1 - Pre-survey for Youth MHFA training

The **Addressing Disparities in Asian Populations through Translational Research (ADAPT)** **Coalition** at the **Tufts Clinical and Translational Science Institute** is conducting a research study to evaluate cultural-responsiveness of Youth Mental Health First Aid (YMHFA) training to challenges facing Asian populations in Greater Boston.

This is the first online survey to be completed BEFORE your scheduled Youth Mental Health First Aide Training.

We ask for your help in conducting this 10-15 minute survey to learn more about your experience BEFORE participating in the Youth MHFA training.

- Participation in the study is voluntary and does not involve any risk. It is YOUR choice whether or not you wish to join the study.
- You can refuse or stop at any time without penalties of any kind
- You may choose not to answer any question, if it makes you feel uncomfortable.

After the MHFA training, we invite you to complete a second online survey. Once both surveys are submitted, you will receive $15 e-gift card.

**We thank you for participation in this study!**

Note: Pre-survey for Adult MHFA training version was similar to the Youth version with the exception of using an adult age (e.g., 30 years old) for vignette part of the Stigma to address mental health issue.

**I. Demographics**

1. What is your race/ethnicity? Select all answers that apply.

 Asian, non-Hispanic

 Asian Indian

 Chinese

 Filipino

 Japanese

 Korean

 Vietnamese

 Other Asian (please specify, for example Hmong, Laotian, Thai, Pakistani, etc.):___________________

 White non-Hispanic

 Black non-Hispanic

 Hispanic, Latino, Latina

 Other non-Asian race: please specify:__________________________

2. Were you born in the United Sates?

 Yes 🡪 Skip to Question 3.

 No 🡪 Go to Question 2a

2a) (Skip question) If you were not born in the United States, how old were you when you moved to the United States?

_________ years old ☐Don’t Know

3. Are you currently at least 18 years old?

 Yes

 No

4. Are you involved with any Asian-serving community-based organization(s) or group(s) in the Greater Boston area?

 Yes -> Go to Question 4a

 No -> Skip to Question 5

 Don’t know -> Skip to Question 5

4a) If yes, please name the organization(s) or group(s): _______________

5. Before this training, have you taken any other Mental Health First Aid Training?

 Yes -> Go to Question 5a

 No -> Skip to Question 6

 Don’t know -> Skip to Question 6

5a) If yes, please state the year you took the training: __________

**II. Self-reported health**

The following questions ask for your views about your health, how you feel and how well you are able to do your usual activities on a typical day. If you are unsure about how to answer a question, please give the best answer you can.

6. In general, would you say your health is

 Excellent

 Very good

 Good

 Fair

 Poor

**III. Literacy on the needs and barriers to mental health care**

Think of someone you know or are close to who is Asian/Asian-American and has or has had a mental health problem. Please refer to this person when you answer the following questions. If you can’t think of anyone, you can refer to yourself.

**Have any of these issues ever stopped, delayed or discouraged this person or you from getting, or continuing with, professional care for a mental health problem?**

*Please check one box on each row to indicate the answer that best suits this person or you.*

|  | **Issue** | This has stopped, delayed or discouraged this person or you  **NOT AT ALL** | This has stopped, delayed or discouraged this person or you  **A LITTLE** | This has stopped, delayed or discouraged this person or you  **QUITE A LOT** | This has stopped, delayed or discouraged this person or you  **A LOT** | This has stopped, delayed or discouraged this person or you  **DON’T KNOW** |
| --- | --- | --- | --- | --- | --- | --- |
| **Cost** | | | | | |  |
| 7. | Not being able to afford the financial costs involved |  |  |  |  |  |
| 8. | Concern that their health insurance (e.g. Mass Health) would not be able to cover the cost |  |  |  |  |  |
| **Knowledge** | | | | | |  |
| 9. | Being unsure where to go to get professional care (e.g. psychologists, psychiatrists, clinical social workers, and counsellors) |  |  |  |  |  |
| 10. | Professionals (e.g. psychologists, psychiatrists, clinical social workers, and counsellors) from his/her own ethnic or cultural group not being available |  |  |  |  |  |
| 11. | Unable to describe or express their mental health issues |  |  |  |  |  |
| **Transportation** | | | | | |  |
| 12. | Having no transportation to help them get professional care (e.g. psychologists, psychiatrists, clinical social workers, and counsellors) |  |  |  |  |  |
| **Personal** | | | | | |  |
| 13. | Wanting to solve things quickly, being discouraged by the speed of progress |  |  |  |  |  |
| 14. | Dislike of talking about their feelings, emotions or thoughts |  |  |  |  |  |
| 15. | Lack of willingness to improve their mental health conditions |  |  |  |  |  |
| 16. | More concern about their family’s basic needs than their own mental health needs |  |  |  |  |  |
| **Stigma** | | | | | |  |
| 17. | Concern about what their family might think, say, do or feel |  |  |  |  |  |
| 18. | Feeing embarrassed or ashamed by community member(s) |  |  |  |  |  |
| 19. | Concern that people they know might find out |  |  |  |  |  |
| **Preference for alternative forms of help** | | | | | |  |
| 20. | Preferring to get help from family or friends |  |  |  |  |  |
| 21. | Preferring to get alternative forms of care (e.g. traditional / religious healing) |  |  |  |  |  |

**IV. Literacy on the mental health help-seeking behavior**

Now think about the same person you know or are close to who is Asian/Asian-American and has had a mental health problem. Please refer to this person as you answer the following questions. If you can’t think of anyone, you can refer to yourself.

**How likely is it that this person or you would seek help from the following people?**

*Please check one box on each row to indicate the answer that best suits this person or you.*

|  | **Not likely** | **Somewhat likely** | **Very likely** | **Don’t Know** |
| --- | --- | --- | --- | --- |
| 22. Relative/family member |  |  |  |  |
| 23. Intimate partner (e.g., girlfriend, boyfriend, husband, wife) |  |  |  |  |
| 24. Friend, colleagues, neighbours (not related) |  |  |  |  |
| 25. Traditional healer |  |  |  |  |
| 26. Mental health professional (e.g., psychologist, psychiatrists, clinical social worker, counsellor) |  |  |  |  |
| 27. Community-based organization / Social service agency |  |  |  |  |
| 28. Phone helpline (e.g., Lifeline) |  |  |  |  |
| 29. Internet support group |  |  |  |  |
| 30. Primary Care Provider/Healthcare Provider |  |  |  |  |
| 31. Minister or religious leader (e.g., Priest, Rabbi, Chaplain) |  |  |  |  |
| 32. Would not seek help from anyone |  |  |  |  |
| 33. If other, please specify: ( ) |  |  |  |  |

The following questions ask about the availability of services in your community.

**What mental health services are provided in your local community?**

*Please check all that you know of and whether these services are linguistically and culturally accessible for Asian populations.*

|  | **Yes** | **If Yes, is this service provided in language(s) accessible to Asian non-English speakers (e.g. Chinese, Vietnamese, Korean, Hindu).**  **(*Leave blank if you don’t know)*** | **If Yes, does this service have culturally-competent providers who have worked with Asian patients before?**  ***(Leave blank if you don’t know)*** |
| --- | --- | --- | --- |
| 34. Mental health professional (e.g., psychologist, psychiatrists, clinical social worker, counselor) |  |  |  |
| 35. Community health center |  |  |  |
| 36. Phone helpline (e.g., Lifeline) |  |  |  |
| 37. Internet support group |  |  |  |
| 38. Minister or religious leader (e.g., Priest, Rabbi, Chaplain) |  |  |  |
| 39. Other (please specify):______________________ |  |  |  |

**V. Stigma to address mental health issue**

The following section concerns a hypothetical youth named Kim. The description below outlines how she has been recently.

**Kim is a 15 year old who has been feeling unusually sad and miserable for the last few weeks. She is tired all the time and has trouble sleeping at night. Kim doesn’t feel like eating and has lost weight. She can’t keep her mind on her work. She puts off making any decisions and even day-to-day tasks seem too much for her. Her friends are very concerned about her. Kim feels like she will never be happy again and believes her family would be better off without her. She has been so desperate, she has been thinking of ways to end her life.**

The next few questions contain statements about Kim’s problem. Please indicate how strongly *YOU PERSONALLY* agree or disagree with each statement.

|  | **Strongly agree** | **Agree** | **Neither agree nor disagree** | **Disagree** | **Strongly Disagree** |
| --- | --- | --- | --- | --- | --- |
| 40. People with problems like Kim could snap out of it if they wanted |  |  |  |  |  |
| 41. A problem like Kim’s is a sign of personal weakness |  |  |  |  |  |
| 42. Kim’s problem is not a real medical illness |  |  |  |  |  |
| 43. People with a problem like Kim ’s are dangerous |  |  |  |  |  |
| 44. It is best to avoid people with a problem like Kim’s so that you don’t develop this problem |  |  |  |  |  |
| 45. People with a problem like Kim’s are unpredictable |  |  |  |  |  |
| 46. If I had a problem like Kim’s, I would not tell anyone |  |  |  |  |  |
| 47. If I had a mental illness like Kim’s, I would not seek help from a mental health professional |  |  |  |  |  |
| 48. I believe treatment by a mental health professional for a mental illness like Kim’s would not be effective. |  |  |  |  |  |

**Now, we would like you to tell us what you think *MOST PEOPLE in the ASIAN/ASIAN-AMERICAN COMMUNITY believe*. Please indicate how strongly you agree or disagree with each statement.**

|  | **Strongly agree** | **Agree** | **Neither agree nor disagree** | **Disagree** | **Strongly Disagree** |
| --- | --- | --- | --- | --- | --- |
| 49. *Most people in the Asian community believe* that people with problems like Kim could snap out of it if they wanted |  |  |  |  |  |
| 50. *Most people in the Asian community believe* that a problem like Kim’s is a sign of personal weakness |  |  |  |  |  |
| 51. *Most people in the Asian community believe* that Kim’s problem is not a real medical illness |  |  |  |  |  |
| 52. *Most people in the Asian community believe* that people with a problem like Kim’s are dangerous |  |  |  |  |  |
| 53. *Most people in the Asian community believe* that it is best to avoid people with a problem like Kim’s so that they don’t develop this problem |  |  |  |  |  |
| 54. *Most people in the Asian community believe* that people with a problem like Kim’s are unpredictable |  |  |  |  |  |
| 55. If they had a problem like Kim’s, *most people in the Asian community* would not tell anyone |  |  |  |  |  |
| 56. If they had a mental illness like Kim’s, *most people in the Asian community* would not seek help from a mental health professional |  |  |  |  |  |
| 57. *Most people in the Asian community believe* treatment by a mental health professional for a mental illness like Kim’s would not be effective. |  |  |  |  |  |

**VI. Mental health literacy**

**For each of the statements below please indicate whether you agree or disagree with it, or don’t know.**

**1) In the general population…**

|  | Agree | Disagree | Don’t know |
| --- | --- | --- | --- |
| 58. Around half of mental disorders starts during childhood or adolescent |  |  |  |
| 59. It is not a good idea to ask someone if they are feeling suicidal in case you put the idea in their head |  |  |  |
| 60. Depression can increase an youth’s risk taking behavior (e.g., reckless driving, risky sexual involvements) |  |  |  |
| 61. People with a psychosis tend to have a better outcome if family members are not critical of them |  |  |  |
| 62. People who harm themselves nearly always want to die |  |  |  |
| 63. Self-harm can be used to help escape from negative feelings such as hopelessness |  |  |  |

***2) In Asian populations***

|  | Agree | Disagree | Don’t know |
| --- | --- | --- | --- |
| 64. Asians are more likely to seek clinical help for mental health illness compared with the general population |  |  |  |
| 65. Suicide is the leading cause of death for Asian youth ages 15-24 |  |  |  |
| 66. Depression is a common mental health illness among Asian population |  |  |  |
| 67. Mental illness in Asian population is caused by weakness of character, poor upbringing or lack of faith |  |  |  |
| 68. Stigam on mental illness discourages Asian community members to seek help |  |  |  |
| 69. US-born Asian Americans experience a higher suicidal thoughts than foreign-born counterparts |  |  |  |

**This is the end of the survey.**

**Thank you!**
