# Supplementary material for: Mental health priorities and cultural-responsiveness of the Mental Health First Aid (MHFA) training for Asian immigrant populations in Greater Boston, Massachusetts
Source: BMC Psychiatry. 2024 Jul 16;24:506. doi: 10.1186/s12888-024-05894-x (PMC11251104; doi:10.1186/s12888-024-05894-x)
Supplement: Supplementary file 2 — Supplementary Material 2 [file 12888_2024_5894_MOESM2_ESM.docx]

Supplemental file 2 - Post-survey for Youth MHFA training

The **Addressing Disparities in Asian Populations through Translational Research (ADAPT)** **Coalition** at the **Tufts Clinical and Translational Science Institute** is conducting a research study to evaluate cultural-responsiveness of Youth Mental Health First Aid (MHFA) training to challenges facing Asian populations in Greater Boston.

This is the second online survey to be completed AFTER your scheduled Youth Mental Health First Aid Training.

We ask for your help in conducting this 10-15 minute survey to learn more about your experience AFTER participating in the Youth MHFA training.

- Participation in the study is voluntary and does not involve any risk. It is YOUR choice whether or not you wish to join the study.
- You can refuse or stop at any time without penalties of any kind
- You may choose not to answer any question, if it makes you feel uncomfortable.

After you complete this survey, you will receive a $15 e-gift card.

We also invite you to participate in the focus group discussion. Once you participated in the focus group discussion, you will receive an additional $15 e-gift card.

**We thank you for participation in this study!**

Note: Post-survey for Adult MHFA training version was similar to the Youth version with the exception of using an adult age (e.g., 30 years old) for vignette part of the Stigma to address mental health issue and including specific sections related to Adult version: 1) ALGEE: Mental Health First Aid Action Plan, 2) MHFA for Early Signs and Symptoms, 3) MHFA for Worsening Signs and Symptoms, and 4) MHFA for Crisis Situation.

**I. Stigma to address mental health issue**

**The following section concerns a hypothetical youth named Kim. The description below outlines how she has been recently.**

*Kim is a 15 year old who has been feeling unusually sad and miserable for the last few weeks. She is tired all the time and has trouble sleeping at night. Kim doesn’t feel like eating and has lost weight. She can’t keep her mind on her work. She puts off making any decisions and even day-to-day tasks seem too much for her. Her friends are very concerned about her. Kim feels like she will never be happy again and believes her family would be better off without her. She has been so desperate, she has been thinking of ways to end her life.*

**The next few questions contain statements about Kim’s problem. Please indicate how strongly *YOU PERSONALLY* agree or disagree with each statement.**

|  | **Strongly agree** | **Agree** | **Neither agree nor disagree** | **Disagree** | **Strongly Disagree** |
| --- | --- | --- | --- | --- | --- |
| 1. People with problems like Kim could snap out of it if they wanted |  |  |  |  |  |
| 2. A problem like Kim’s is a sign of personal weakness |  |  |  |  |  |
| 3. Kim’s problem is not a real medical illness |  |  |  |  |  |
| 4. People with a problem like Kim’s are dangerous |  |  |  |  |  |
| 5. It is best to avoid people with a problem like Kim’s so that you don’t develop this problem |  |  |  |  |  |
| 6. People with a problem like Kim’s are unpredictable |  |  |  |  |  |
| 7. If I had a problem like Kim’s, I would not tell anyone |  |  |  |  |  |
| 8. If I had a mental illness like Kim’s, I would not seek help from a mental health professional |  |  |  |  |  |
| 9. I believe treatment by a mental health professional for a mental illness like Kim’s would not be effective. |  |  |  |  |  |

**II. Mental health literacy**

**For each of the statements below please indicate whether you agree or disagree with it, or don’t know.**

**1) In the general population**

|  | Agree | Disagree | Don’t know |
| --- | --- | --- | --- |
| 10. Around half of mental disroders starts during childhood or adolescent |  |  |  |
| 11. It is not a good idea to ask someone if they are feeling suicidal in case you put the idea in their head |  |  |  |
| 12. Depression can increase an youth’s risk taking behavior (e.g., reckless driving, risky sexual involvements) |  |  |  |
| 13. People with a psychosis tend to have a better outcome if family members are not critical of them |  |  |  |
| 14. People who harm themselves nearly always want to die |  |  |  |
| 15. Self-harm can be used to help escape from negative feelings such as hopelessness |  |  |  |

**2) In Asian populations**

|  | Agree | Disagree | Don’t know |
| --- | --- | --- | --- |
| 16. Asians are more likely to seek clinical help for mental health illness compared with the general population |  |  |  |
| 17. Suicide is the leading cause of death for Asian youth ages 15-24 |  |  |  |
| 18. Depression is a common mental health illness among Asian populations |  |  |  |
| 19. Mental illness in Asian populations is caused by weakness of character, poor upbringing or lack of faith |  |  |  |
| 20. Stigma on mental illness discourages Asian community members to seek help |  |  |  |
| 21. US-born Asian Americans experience a higher suicidal thoughts than foreign-born counterparts |  |  |  |

**III. Feedback about Mental Health First Aid Training**

*Next, we will ask you questions about your experience in the Youth Mental Health First Aid (YMHFA) Training*

22. Which section(s) in the YMHFA training were informative for addressing mental health issues among Asian populations? (Select all that apply):

 None of the sections

 YMHFA in Non-crisis Situations

 YMHFA for Crisis Situations

 Self-care for the Youth Mental Health First Aider

22a. If you checked any boxes above, please describe how so:

______________________________________________________________________

23. During the YMHFA training, were there any specific examples or case studies that were tailored to Asian populations?

 No

 Yes, 1-2 examples or case studies

 Yes, 3-5 examples or case studies

 Yes, 6 or more examples or case studies

24a. If you selected Yes, please describe these examples or case studies: _____________________________________________________________________

24. Do you have feedback on how to the YMHFA training can be more *culturally-responsive* to mental health issues in the Asian communities?

25. Which section(s) in the YMHFA training were informative for addressing mental health issues among immigrant or refugee populations? (Select all that apply):

 None of the sections

 YMHFA in Non-crisis Situations

 YMHFA for Crisis Situation

 Self-care for the Youth Mental Health First Aider

25a. If you checked any boxes above, please describe how so:

______________________________________________________________________

26. During the YMHFA training, were there any specific examples or case studies that were tailored to immigrant and refugee populations?

 No

 Yes, 1-2 examples or case studies

 Yes, 3-5 examples or case studies

 Yes, 6 or more examples or case studies

26a. If you selected Yes, please describe these examples or case studies:

_____________________________________________________________________

27. Do you have feedback on how to the YMHFA training can be more culturally-responsive to mental health issues in the immigrant and refugee communities?

**IV. Literacy on the needs and barriers to mental health care**

28. Did the Youth Mental Health First Aid training that you just completed help you recognize any of the following potential barriers to individuals seeking mental health care? (Select all that apply):

 Financial costs

 Lack of insurance coverage

 Lack of knowledge on where to get the professional care (*such as from psychologists, psychiatrists, clinical social workers, counsellors)*

 Lack of professionals (*e.g.* *psychologists, psychiatrists, clinical social workers, counsellors*) from individual’s own ethnic or cultural group

 Inability to describe or express one’s mental health issues

 Lack of transportation

 Slow progress of mental health care

 Dislike of talking about one’s feelings, emotions or thoughts

 Unwillingness to improve

 Concern about stigma from family member(s)

 Feeling embarrassed or ashamed by community member(s)

 Concern that people might find out

 Preference to only solve the mental illness by oneself

 Preference to only seek help from family or friends

 Preference to only seek alternative forms of help (e.g. traditional / religious healing)

 Fear of being institutionalized to mental health facilities

 Fear of medication side effects

 Lack of internet access for telemedicine

 Negative experience with previous professionals (*e.g.* *psychologists, psychiatrists, clinical social workers, counsellors*)

 Other barriers, please specify: _____________________

29. Are there any other topics that you wish were covered during the YMHFA training?

__________________________________________________________________________

30. What other language(s) should the YMHFA training be provided in?

 Chinese – Mandarin

 Chinese – Cantonese

 Filipino

 Japanese

 Korean

 Vietnamese

 Hindi

 Other (please specify): ____________________

31. Would you recommend the YMHFA training to another community member?

 Yes

 No

 Not sure

32. What resource(s) would be helpful for you to know/have when responding to individuals with mental health issues in your community? (Select all that apply):

 Phone helpline

 Online support groups

 In-person support groups

 Recreational activities (e.g. sports, dance, cooking classes)

 Cultural programming (e.g. dance, arts, music, theater)

 Books or reading materials

 Directory of culturally-competent mental health professionals (e.g., psychologist, psychiatrists, clinical social worker, counselor)

 Directory of mental health professionals (e.g., psychologist, psychiatrists, clinical social worker, counselor) that speak an Asian ethnic language (e.g. Chinese, Vietnamese, Korean, Hindu)

 Directory of ministers or religious leaders (e.g., priest, rabbi, chaplains)

 Directory of community health centers

 Other, please describe: __________________________________________________

**This is the end of the survey.**

**Thank you!**
