# Supplementary material for: Mental health priorities and cultural-responsiveness of the Mental Health First Aid (MHFA) training for Asian immigrant populations in Greater Boston, Massachusetts
Source: BMC Psychiatry. 2024 Jul 16;24:506. doi: 10.1186/s12888-024-05894-x (PMC11251104; doi:10.1186/s12888-024-05894-x)
Supplement: Supplementary file 3 — Supplementary Material 3 [file 12888_2024_5894_MOESM3_ESM.docx]

Supplemental Table 3 - Thematic coding and quotes from focus group with staff (N=10)

| **Theme** | **Subtheme** | **Quotes** |
| --- | --- | --- |
| 1. Common mental health issues | Loneliness | "We're hearing a lot about isolation, feeling lonely, among mostly elders as well as older adults who are kind of home bound during COVID" |
|  | Depression | "A lot of [Asian American youth] are very high functioning when they have depression" |
|  | Anxiety (social and financial difficulty) | "Due to rising cost of living and material condition, I notice a lot of the anxiety among clients about their finances and rent" "Parents have anxiety because their kids are not socializing and social anxiety is something that I'm noticing as well because of again COVID effect" |
|  | Trauma (immigration, generational) | "People whose parents were immigrants have that survival mentality, and I think that feeds into their children having a lot of anxiety, having depression, being unable to navigate the world" "Undocumented and some of the immigrants, they deal with a lot of trauma coming here to the US" |
|  | Acculturation stress | "Acculturation stress and how this has been identified as a factor that's associated with negative mental health outcomes like depression" |
| 2. Mental Health Challenges and Priorities | COVID-19 pandemic (home confinement, social isolation, Anti-Asian racism, violence against Asians, Travel restrictions) | "The anti-Asian hate crimes for the past couple of years, a lot of them is just terrified" |
|  | Lack of access to language-appropriate resources | "Access to mental health professionals, especially on language level, a lot of them couldn't find the kind of professionals who speak their own languages" |
|  | Cultural difference between American and Asian (parent-child communication, difficulty reaching for mental health issues, gender expectations, lack of personal space, conflicting environment between home and school) | "It's hard for them to talk about it with their parents because their parents don't understand"  "Even the topic of talking about mental health and talking about therapy is also difficult, too, because they don't fully understand like what that means or how to make their children feel loved and supported.” |
|  | Intentional negligence (deferring mental health care for other priorities, long waitlist for therapy) | "Waitlist for getting therapy is just so ridiculously long" "We often have people coming and saying we don't have time to for this, we're busy working just to feed our family" |
|  | Unintentional negligence (unawareness of mental distress) | "They're just not even aware that they are going through this mental health issue and identify it as such" |
|  | Limited mental health education (social emotional learning) | "The general public would like to have the vocabulary necessary to describe what they're feeling, how they're feeling" |
|  | Lack of physical, recreational spaces to relieve mental distress | "It's like not having a lot of space where they feel like they belong" "You know [Asian Americans adults] are not going to go to a bar or have drinks with their friends. They want to go to karaoke or playing ping pong or something like that" |
| 3. Mental health seeking behavior and barriers | Indirect approach (via engagement in family services, afterschool programs) | "It's very rare, at least based on my anecdotal experience, that clients come specifically to seek mental health assistance. It's usually something that comes up in the discussion about other things [like family service, afterschool program]" |
|  | Informal methods (online search, friends, religious leaders) | "When people, especially older generation, trouble talking to their family, what they do is they see their monks or their bishops as their mental health or spiritual leader" |
|  | Stigma (worry about other members finding out, model minority myth) | "One thing that's unique in the Asian community is that we put less emphasis on individual than the fact we treat family as a unit, so there's really not much privacy per say" "In the Asian community, mental health needs and distress are easily brushed off as a flaw of character" |
|  | Poor understanding about mental health services (limited language about mental health resources, mental health vocabularies, lack of trust in mental health system) | "Definitely seeing a need of sort of understand the nature of mental health, even social emotional learning and what it can be done" |
|  | Immigration status (undocumentation) | "I've heard that a lot of undocumented folks are really afraid of seeking service, and when they are seeking services, it's still hard for the provider to really build trust quickly, because [undocumented folks are] kind of afraid that things they get into or the services they seek will get them into trouble and I think that's another thing to keep in mind." |
|  | Financial barrier (high cost, prioritizing basic needs first) | "You have to worry about your rent, your utilities, and your food, it's hard to budget out enough money for therapy" |
|  | Language barrier (lack of Asian language speaking counselors, lack of bilingual translators) | "There was a bilingual guidance counselor who wasn't even my guidance counselor, was just another person in the office that happened to speak Chinese. She was able to kind of direct my mom to [mental health] services when we needed so it was kind of like a lucky situation." |
| 4. Training needed to support Asian community members with mental health issues | Communication (appropriate description of mental distresses, intergenerational dialogue between parents and children) | "With their communication with the parents, who, if they are new immigrants, it's harder for them to be on the same page, or even knowing how to respect the cultural difference or the cultural identity and how we find a middle point" |
|  | Training workforce (community health workers, providers, and staffs) | "A lot of the trainings I received I feel like were built for predominantly White culture" "I wish I could just get training from someone who works primarily with [Asian] population" |
| 5. Education needed to support Asian community members with mental health issues | Health system in the US (Insurance coverage/payment sources, community health centers) | "Trainings or education about the systems in the America could also help. A lot of adults sort of understand what are their resources and how they can ask for help with different communities and with different programs, different services" |
|  | Systematic issues (model minority myth, unrealistic expectation, oppression) | "I think it's important to have some type of training or education for the community about systemic issues in America, the injustices that's happening to kind of break the myth, like the model minority myth" |
|  | Navigating culturally-responsive mental health resources (comprehensive guide to available resources in the community) | "More education on where they can find culturally responsive services and treatment and support" |
|  | Basic knowledge about mental health issues (types, medications, symptoms, terminologies) | "If there was a Chinese version of this, where people could be aware of what those terms were, it would be really helpful" |
|  | Destigmatizing effort | "The most important or most helpful thing I think we can do is to frame it so people don't feel stigmatized when they need to seek mental health care" |
| 6. Social contexts to consider for Asian community members with mental health issue | Mindful of Asian traditional beliefs, religions, and medical practice, cultural background | "I think we're a very observant community where we're observing, we're waiting that right moment where we feel comfortable to seek help" |
|  | Closely knit community (lack of trust, lack of privacy, high judgment) | "I have worked with clients who are very concerned that their problems that they talk to us will spread to the community the next day" "The invisible judgement I get asked quite often like, is my neighbor going to see me talking to you?" |
|  | Variation among Asian ethnic groups (Eastern, South) | "Different ethnic groups go to different treatment center or community health centers" "We see a lot of PTSD passed on from generation to generation, particularly in the Cambodian or Vietnamese communities" |
|  | Incongruence with standardized language (need glossary for translation, lost in translation) | "I've had someone who actually told me about how her lungs are having too much heat. In [Asian] medicine, I think that's relating to anxiety" "Some language doesn't even have a word to describe different mental illness" |
|  | Stress and burdens that immigrants experience (trauma, outsider/foreigner treatment) | "Not having the life they used to have just because they moved to a different country, it's sort of something that's an ongoing struggle for them" "They've kind of built this hard shell and like a bunch of onion peels they cannot feel their emotions and they can't take about how they affect them even when they talk about their immigration stories" |
|  | Marginalized groups (non-heterosexual orientations, elders, etc.) | "For a lot of gay and trans folks that are within the Asian community, they might face getting kicked out [of their homes]" "Asian elders usually have a hard time to understand the model of talk therapy" |
