# Supplementary material for: Mental health priorities and cultural-responsiveness of the Mental Health First Aid (MHFA) training for Asian immigrant populations in Greater Boston, Massachusetts
Source: BMC Psychiatry. 2024 Jul 16;24:506. doi: 10.1186/s12888-024-05894-x (PMC11251104; doi:10.1186/s12888-024-05894-x)
Supplement: Supplementary file 4 — Supplementary Material 4 [file 12888_2024_5894_MOESM4_ESM.docx]

Supplemental table 4 - Thematic coding and quotes from focus group with community participants (N=8)

| **Theme** | **Subtheme** | **Quotes** |
| --- | --- | --- |
| 1. Common mental health issues | Academic stress | "They are very stressful when they face the major examinations, especially in the university" |
|  | Depression | "I think in general, depression and anxiety are the most common amongst students for many college campuses" |
|  | Anxiety | "I wouldn't see people's faces and they couldn't see mine and hopefully that they wouldn't see my disorder" |
|  | Loneliness | "[International students] just study alone here, so they would feel like they missed their family" |
|  | Attention-deficit/hyperactivity disorder (ADHD) | "A lot of symptoms of ADHD, they'll vent to me about these things 'I have such a hard time sleeping. I have a hard time focusing'" |
|  | Generational trauma | "Very often I feel that parents are projecting their life values, such as becoming a doctor or becoming a lawyer or getting married, and these projecting of life values can have a huge toll on the children" |
| 2. Mental Health Challenges and Priorities | Difficulty fitting in and making friends (social connection, loneliness, lacking connectedness) | "[Asian youth] don't feel connected with their friends or someone who's really connected to them" |
|  | Substance use (marijuana) | "[My daughter] said students would use [marijuana] and would invite them to try to it, so sometimes it's very difficult when you say no" |
|  | Peer and familial pressure | "The parents would say, 'Why you feel depressed, you already got everything'" |
|  | Confiding personal issues to "strangers" | "She doesn't feel comfortable seeing a therapist because it's talking to someone she doesn't know, and she preferred to talk about really personal, intimate, like mental health, issues with someone she knows" |
|  | Asian hate crime | "It was a struggle for the community overall, for Asian-owned businesses and the protection, security, just walking out, going grocery shopping or even having their store open" |
|  | Accessibility to mental health services | "Every time we make a referral, they will say, 'Oh, the waiting is at least for six months or even more than a year,' so it is very challenging for them to get services right away" "Our clients, they can only speak Mandarin and Cantonese. Although they can understand limited English, it is very difficult for them to find other resources by themselves" |
|  | Mental health literacy (Connection to resources, knowledge about mental disorders) | "A lot of times, it's framed black and white, where you're either in crisis or not, and if you're not, you don't need help" "A lot people weren't talking about [mental health]. I wasn't even aware that a lot of these things, a lot of these topics existed" "A lot of people kind of undermine the importance of taking care of one's mental health" |
|  | Social/structural factors (cliff effect, job insecurity, social benefit, housing insecurity) | "We're kind of stuck on being poor all the time in order to be on MassHealth so we can pay, we can get medical coverage, our medication, see our therapists, our doctors" |
|  | Support group | "Support groups and skill-based groups that can come together and discuss self-help resources can be very helpful" |
|  | Community-driven interventions and services | "They will come to our organization to see whether there's other opportunity for them to get resources or get services" |
| 3. Most useful/most relevant part of MHFA training for Asians | ALGEE (Approach assess for risk of suicide or harm and assist, Listen non judgmentally, Give reassurance in information, Encourage appropriate professional help, Encourage self-help and other support strategist) | “Learning about ALGEE plan helps me give background knowledge on the things I can do if I were in the situation of providing mental health support” |
|  | Self-paced lesson prior to training | “The self-pace area where it covers a lot of psychoeducation information, like 1 out of 5 youth in the state actually suffer from mental challenges and different types of disorders, like anxiety and depression. That was really helpful because I didn’t know it’s so common” |
|  | Case studies featuring Asians | “What I see in that Chinese family — they look like they’re first generation and the son can speak Chinese but also speaks English most of the time, and he’s pretty much Americanized — I see in him is the father reacts just like my parents” |
|  | Nonjudgement about mental health issues (taking time, not pushing to make decision) | “To not push the person you are trying to help and the cultural consideration part is very helpful, especially for working with aging adults or youth” |
|  | Feeling more confident about referring and encouraging professional help among Asians | “After the training course, I think I am more confident to discuss [reaching out for professional help] with [a friend struggling with mental health]” |
|  | Norms/values/common trends among Asian communities when it comes to mental health (e.g., high expectations/high functioning) | “There’s very high stigma in Asian culture. Even recommending therapists is like something’s really wrong with you” |
| 4. Least useful/least relevant and recommendations for future MHFA training to be more culturally-responsive for Asians | Lack of learning execution skills (conversation, emphasizing with speaker) | "I thought it would be useful to talk more about the stigma and how to have those conversations with different generations or people with very different views" |
|  | Asian language options | "Although they can speak English, speaking in their primary language I think would be totally different experiences for them. They will feel more comfortable and they can share more about their stories" |
|  | Lack of culturally-responsive case studies/examples (family dynamic, immigrant status, academic stress) | "I think the MHFA training did not touch a lot upon specifically-Asian or being culturally sensitive" "It was not clear if there was any cultural effect being an obstacle between this person from improving their mental health" "I think definitely the case study scenario discussion can include more cultural awareness, for example, based on new immigrants or undocumented immigrants"  "I do think it would be nice if we had like more on like dealing with academic stress and intergenerational struggle" |
|  | Short training hours | "I prefer a workshop series" "There was lots of different concepts for one day" "I was going through almost 8 hours on Zoom" |
|  | More focus on helpful and unhelpful responses to mental health | "At the beginning of our mental health training, we went through states that were helpful, like 'Oh, I have been through similar issues' or 'You'll get over it,' like common phrases used to brush off mental health challenges" |
| 5. Asian community that can benefit from MHFA training | School (counselor, advisors, teachers, program leaders, professors) | "Since professors and those in academia have more interactions with the students, they have the opportunity to know the students in a more one-on-one as well as to cultivate learning and to be a support system for students" |
|  | LGBTQIA+ community | "I think the gender issue is very hard for kids to tell their parents in Asia" |
|  | Service providers (youth centers, therapists, police, social workers) | "Social workers definitely are handling certain minority groups or Asian groups, so they can have them talk to the right people" "Policemen definitely need to have somebody in there to take care of this first part to identify, he or she is not a threat to us, and we recognize that it was a panic attack" |
|  | Parents, elders | "I noticed that there are several parents signed up for the mental health training because they want to know more about how to interact with their teens and also want to support them" |
|  | Religious leaders | "Within the Asian community, they may feel more comfortable going to someone within the same faith or same community instead of going to a mental health professional" |
