# Supplementary material for: Mental health priorities and cultural-responsiveness of the Mental Health First Aid (MHFA) training for Asian immigrant populations in Greater Boston, Massachusetts
Source: BMC Psychiatry. 2024 Jul 16;24:506. doi: 10.1186/s12888-024-05894-x (PMC11251104; doi:10.1186/s12888-024-05894-x)
Supplement: Supplementary file 5 — Supplementary Material 5 [file 12888_2024_5894_MOESM5_ESM.docx]

Supplemental table 5a – Additional Feedback about the **Youth** MHFA training from the post-training questionnaires (N=21)

|  | **Percentage (%)** |
| --- | --- |
| Sections in the MHFA that were informative for addressing mental health issues among Asian populations | |
| None | 14% |
| YMHFA in Non-crisis Situations | 81% |
| YMHFA for Crisis Situations | 76% |
| Self-care for the Youth Mental Health First Aider | 67% |
| Qualitative Feedback:  "It clarified proper ways to engage people who are going through mental health issues and knowing the appropriate steps to take and what specific situations call for. I think there was a lot of stress on taking care of the self as well, which is very important in helping youth."  "Now I would talk with the youth and reach out for referrals. Having a self-care plan is very important as this has not been brought up in my upbringing. I might have done too much and expanded my boundaries until I feel burned out. I'd also like to share it to students too." | |
| Number of examples or case studies that were tailored to Asian populations | |
| No | 62% |
| Yes, 1-2 examples or case studies | 33% |
| Yes, 3-5 examples or case studies | 5% |
| Yes, 6 or more examples or case studies | 0% |
| Qualitative Feedback:  "Statistics on suicide rates and how unlikely it is for Asian youth to seek mental healthcare. "  "The video included an Asian person struggling with anxiety following a concussion."  "I am not sure but one of the case studies featured a young man that may have been Asian." | |
| How can the YMHFA training can be more culturally-responsive to mental health issues in the Asian communities? | |
| Qualitative Feedback:  "I think getting more context as to why folks might be resistant to seeking help in regard to Asian cultures. I think more stress on not placing the blame on the individual links to that."  "Adding more pie charts/ graphs that have the stats labelled. It can help to show the magnitude of the bad effects of mental health crisis is relative to other adversity faced by the Asian community." | |
| Sections in the MHFA that were informative for addressing mental health issues among immigrant or refugee populations | |
| None | 57% |
| YMHFA in Non-crisis Situations | 29% |
| YMHFA for Crisis Situations | 33% |
| Self-care for the Youth Mental Health First Aider | 24% |
| Qualitative Feedback:  "I think we addressed trauma, but not necessarily naming specific kinds of traumas that immigrants/refugees go through."  "Nothing specifically that jumped out at me." | |
| Number of examples or case studies that were tailored to immigrant and refugee populations | |
| None | 86% |
| Yes, 1-2 examples or case studies | 14% |
| Yes, 3-5 examples or case studies | 0% |
| Yes, 6 or more examples or case studies | 0% |
| Qualitative Feedback:  "Immigrants are as likely to be diagnosed with depression or anxiety."  "The training didn't be tailored to the immigrant or refugee population." | |
| Do you have feedback on how to the YMHFA training can be more culturally-responsive to mental health issues in the immigrant and refugee communities? | |
| Qualitative Feedback:  "Perhaps including examples from those communities."  "Learning more about the history about those populations and why it may affect mental health."  "Show specific cases about immigrant/refugee individuals." | |
| Are there any other topics that you wish were covered during the YMHFA training? | |
| Qualitative Feedback:  "How to provide psychoeducation about seeking professional help."  "How to deal with a person who refuses help. How to deal with a parent who refuses help for their child."  "I think it may be useful to run through scenarios where you may speak with parents or family as well." | |

Supplemental table 5b – Feedback about the **Adult** MHFA training from the post-training questionnaires (N=3)

|  | **Percentage (%)** |
| --- | --- |
| Sections in the MHFA that were informative for addressing mental health issues among Asian populations | |
| None | 0% |
| ALGEE: Mental Health First Aid Action Plan | 67% |
| MHFA for Early Signs and Symptoms | 67% |
| MHFA for Worsening Signs and Symptoms | 67% |
| MHFA for Crisis Situation | 67% |
| Self-care for the Mental Health First Aider | 100.0% |
| Qualitative Feedback:  "The ALGEE Plan gave a general guideline of how to support someone. The MHFA early signs and symptoms, worsening signs and symptoms and crisis gave a general idea of what to be aware of and how to handle various situations as well as emphasizes on not diagnosing someone, but to provide resources and support." | |
| Number of examples or case studies that were tailored to Asian populations | |
| No | 33% |
| Yes, 1-2 examples or case studies | 33% |
| Yes, 3-5 examples or case studies | 33% |
| Yes, 6 or more examples or case studies | 0% |
| Qualitative Feedback:  "The boxing video with the coach and aunt definitely portrayed a possible Asian population case study. The video showed the pressures and degrading approach that families can add to the child's life. These examples indicate that the tone of voice and anger approach can elevate to a crisis. Breaking down the helpful and unhelpful steps and finding ways to resolve the situation that can be tailored to Asian populations." | |
| How to the MHFA training can be more culturally-responsive to mental health issues in the Asian communities? | |
| Qualitative Feedback:  “Discuss the concepts of shame, respect, and filial piety in embedded in Asian culture and how they affect Asians at different stages in life.”  “More example relating to Asian Communities - more information/statistics - possibly breaking down the data more such as specifying ethnic groups.” | |
| Sections in the MHFA that were informative for addressing mental health issues among immigrant or refugee populations | |
| None | 67% |
| ALGEE: Mental Health First Aid Action Plan | 33% |
| MHFA for Early Signs and Symptoms | 33% |
| MHFA for Worsening Signs and Symptoms | 33% |
| MHFA for Crisis Situation | 33% |
| Self-care for the Mental Health First Aider | 100% |
| Qualitative Feedback:  "Normally when it comes to immigrants and refugees, there are language barriers, costs can be stressful, access to insurance, and being in a new country there's also that fear. Synthesizing the sections and breaking it down was an effective way to strategize how to de-stigmatize, encourage others their approach." | |
| Number of examples or case studies that were tailored to immigrant and refugee populations | |
| No | 67% |
| Yes, 1-2 examples or case studies | 33% |
| Yes, 3-5 examples or case studies | 0% |
| Yes, 6 or more examples or case studies | 0% |
| Qualitative Feedback:  "The chapter on ALGEE- Mental health first and action plan, discussing how culture intersects with our communication methods was very essential." | |
| Do you have feedback on how to the MHFA training can be more culturally-responsive to mental health issues in the immigrant and refugee communities? | |
| Qualitative Feedback:  “We watched a few videos, perhaps ask the trainees if they have any similar experiences that they are willing to share and how they resolved a scenario.” | |
| Are there any other topics that you wish were covered during the MHFA training?  Qualitative Feedback:  "Expand more on what it means to be culturally sensitive and how to ask questions regarding how someone can be of support." | |
